# Compensation of capacitive currents in high-throughput dielectrophoretic separators

**DOI:** 10.1038/s41598-024-67030-9

**Published:** 2024-07-17

**Authors:** Jasper Giesler, Laura Weirauch, Jorg Thöming, Michael Baune

**Affiliations:** 1 Chemical Process Engineering, Faculty of Production Engineering, University of Bremen, Leobener Straße 6, 28359 Bremen, Germany; 2 MAPEX Center for Materials and Processes, University of Bremen, 28359 Bremen, Germany; 3 Center for Environmental Research and Sustainable Technology (UFT), University of Bremen, Leobener Straße 6, 28359 Bremen, Germany

**Keywords:** Chemical engineering, Applied physics

## Abstract

Separation and classification are important operations in particle technology, but they are still limited in terms of suspended particles in the micrometer and nanometer size-range. Electrical fields can be beneficial for sorting such particles according to material properties. A mechanism based on strong and inhomogeneous fields is dielectrophoresis (DEP). It can be used to separate microparticles according to their material properties, such as conductivity and permittivity, by selectively trapping one particle type while the other can pass the separator. Conventional DEP-separators show either a limitation in throughput or frequency bandwidth. A low throughput limits the economical feasibility in many cases. A lower frequency bandwidth limits the variety of materials that can be sorted by DEP. To separate semiconducting particles from a mixture containing particles with higher conductivity according to their material, high frequencies are required. Possible applications are the separation of semiconducting and metallic carbon nanotubes or the separation of carbon-coated lithium iron phosphate particles from graphite in the recycling process of spent lithium-ion batteries. In this publication, we aim to display how to tune the electrical impedance of a high-throughput DEP separator based on custom-designed printed circuit boards to increase its frequency bandwidth. By adding inductors to the electrical circuit, we were able to increase the frequency bandwidth from 500 kHz to over 11 MHz. The experiments in this study act as proof-of-principle. Furthermore, a non-deterministic way to increase the impedance of the setup is shown, yielding a maximum frequency of 39.16 MHz.

## Introduction

The separation of particle mixtures with dimensions in the range of a few micrometers or even nanometers is currently the subject of research^1–3^. This is due to the fact that important separation tasks involve microparticles, such as separating cell mixtures^4,5^ or separating black mass when recycling lithium-ion batteries^1,6^. In addition, as particle size decreases, separation forces as inertia become smaller since their effects are increasingly compensated by diffusion^7,8^.

Various separation methods for microparticles are already being applied. To name a few, field-flow fractionation^9^, (gel-) electrophoresis^10^, density gradient centrifugation^11^, aerodynamic lenses^12^ or size-exclusion chromatography^13^ are prominent examples. These methods definitely have their benefits, but they also have their drawbacks, such as either low throughput, high costs, the need of charges on the particles or lack of scalability. Depending on the characteristics of the particles to be separated, these disadvantages can limit their applicability significantly.

Dielectrophoresis (DEP) is another technique for separation problems involving (sub)micrometer-sized particles. The separation can be size-^14–16^, shape-^17^, or material-selective^18^. The size selectivity can be derived from the volume dependence of the dielectrophoretic force $\textbf{F}_\text{DEP}$^19^:

1 $$\begin{aligned} \textbf{F}_\text{DEP}=2\pi \varepsilon _m\text{Re}(\tilde{f}_{\text{CM}})r_\text{p}^3\nabla \mathbf {E_{rms}}^2. \end{aligned}$$

here the electric field is denoted as $\mathrm {{\textbf {E}}_{rms}}$, the radius of the particle (subscript p) as $r_\text{p}$, the permittivity of the medium (subscript m) as $\varepsilon _\text{m}$, and $\text{Re}(\tilde{f}_{\text{CM}})$ is the real part of the so-called Clausius–Mossotti factor. This factor can either be positive, negative or zero, depending on the properties of the particle and the surrounding medium. For a spherical and homogeneous particle, it is commonly written as^20^

2 $$\begin{aligned} \text{Re}(\tilde{f}_{\text{CM}})=\text{Re}\left( \frac{\tilde{\varepsilon }_\text{p}-\tilde{\varepsilon }_\text{m}}{\tilde{\varepsilon }_\text{p}+2\tilde{\varepsilon }_\text{m}} \right) . \end{aligned}$$

**Figure 1:**
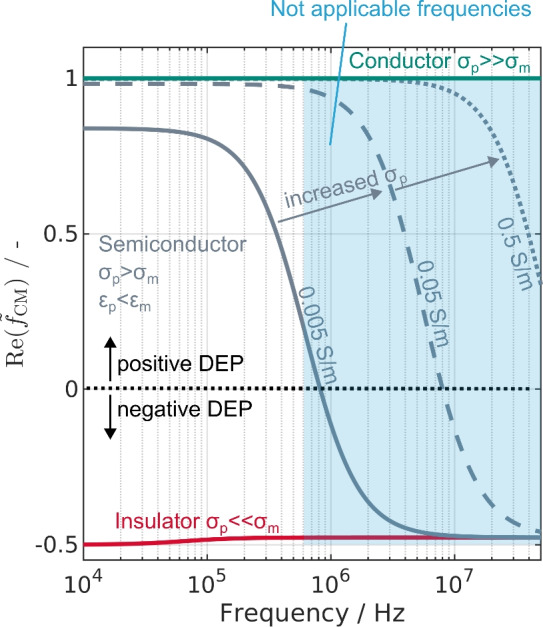
Calculated real-part of the Clausius–Mossotti factor ($\text{Re}(\tilde{f}_{\text{CM}})$) over frequency for different particle conductivities. Assumed medium conductivity is σ_m_ = 3 μS/cm and 80 for the relative permittivity. Particles have a relative permittivity of 2.5 in the calculations and conductivites of 1e−12 S/m (insulator), 0.005 S/m, 0.05 S/m, 0.5 S/m (semiconductors), and 1e6 S/m (conductor). Additionally, the range of not-applicable frequencies in the previously presented setup is highlighted in blue. This figure is based on Ref.^24^.

The complex permittivity $\tilde{\varepsilon }=\varepsilon -i\sigma /\omega$ includes not only the permittivity but also the angular frequency of the electric field $\omega$ and the conductivity $\sigma$ of a material. When the particle is more polarizable than the surrounding medium (e.g., $\sigma _\text{p}\gg \sigma _\text{m}$) $\text{Re}(\tilde{f}_{\text{CM}})$ is positive and $\textbf{F}_\text{DEP}$ is directing the particle towards a local field maxima (Fig. 1 green line). This is called positive DEP (pDEP). Negative DEP (nDEP), in contrast, is pushing particle away from high field regions and occurs for particles with lower polarizability than the medium (Fig. 1 red line). Both, pDEP and nDEP can be used to separate particles, for example by using dielectrophoretic field-flow-fractionation^21^ or trapping of particles in high field regions^22,23^.

From Eq. 2 one can see the interplay of permittivity, conductivity and frequency which allows adjusting the direction of the dielectrophoretic force by changing the frequency. This is, however, only possible for certain combinations of medium and particle. When the particle has higher conductivity and permittivity compared to the medium, no crossover from pDEP to nDEP is expected. In contrast, for semiconducting particles with a higher conductivity but lower permittivity compared to the medium (e.g., pure water), a crossover can be observed when increasing the frequency (Fig. 1 gray lines). With water having a higher relative permittivity than many particles, material-selective separation could be used to separate two semiconducting particles from one another or a semiconducting particle from a conductor. This could be achieved by selecting a frequency where one particle shows pDEP and gets retained in a separator whereas the other, with lower conductivity, shows nDEP and gets eluted. Interesting particle mixtures that contain semiconducting microparticles and could be separated in this way are, for example, carbon-coated lithium iron phosphate, as it is used in lithium-ion batteries^25–27^, metallic and semiconducting carbon nanotubes^28–30^ or rare earth minerals^31–33^.

One option to cause a semiconductor to show nDEP behavior is to increase medium conductivity to reduce the crossover frequency. However, this comes at the cost of increased thermal flows^34^. Although some concepts utilize hydrothermal flows for manipulation of (bio-)particles^35^, these can significantly interfere with DEP trapping in electrode-based processes^8,34^. Alternatively, as can be seen from Fig. 1, high frequencies can be used to achieve a crossover from pDEP to nDEP for a semiconductor. For microfluidic devices, where DEP is commonly applied, the use of high frequencies has been reported numerous times^3,36–38^. Recently, significant efforts were put into the upscaling of DEP separators that maintain the capabilities of microfluidic devices to increase the otherwise limited throughput of DEP separators^22,23,39–41^.These upscaled separators are, unfortunately, limited in their frequency bandwidth because larger separators require higher voltages and/or currents to achieve sufficient electric field strength to manipulate particles via DEP.

### The problem

For insulator-based (iDEP) and electrode-based (eDEP) upscaled DEP separators (volume flow >100 ml/h), we have found that even state-of-the-art amplifiers cannot provide sufficient voltage or current for these setups at high frequencies. The reason for this is that technical amplifiers have a limited slew rate^46^ [volts per second and amperes per second] at which they can change the signal. This slew rate can be exceeded if the frequency is increased. In order to describe this major limitation of the applicability of DEP separators, a suitable model for describing their impedance is required. The total current $I_\text{tot}$ flowing through an area *A* (e.g., an electrode array) can be calculated with[^42^, Equations 2.40 and 2.51]

3 $$\begin{aligned} { I_\text{tot}=\iint _A \left( \sigma _\text{m} \textbf{E}+\varepsilon _\text{m}\frac{\partial \textbf{E}}{\partial t}\right) \cdot dA.} \end{aligned}$$

The current has a part that is proportional to the conductivity of the medium (free current) and the so-called displacement current ($\varepsilon _\text{m}\partial \textbf{E} / \partial t$). It is frequency-dependent as it is proportional to the time derivative of the electric field. Following these equations, the current is expected to increase with increasing frequency, which is well known, for example, for capacitors, but also was observed in our group during experiments with DEP separators.

**Figure 2:**
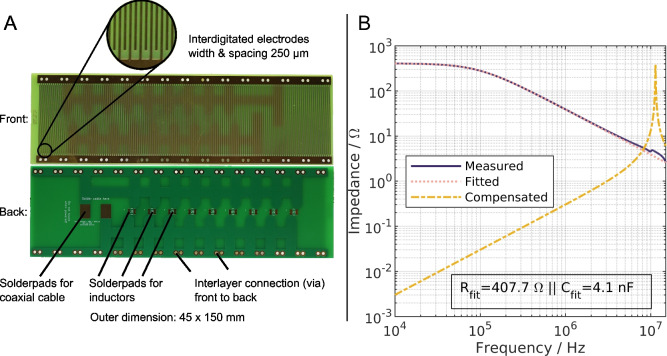
(**A**) Photographs of the printed circuit boards (PCBs) used in this study. The PCBs have an electrode array on the front (width and spacing both are 250 μm). On the back, solder pads for coaxial cable and inductors are located. A coaxial cable is used to connect the channel to the amplifier and vector network analyzer. Inductors are used for the compensation of capacitive currents. Front and back of the PCBs are connected by interlayer connections. (**B**) Measured absolute impedance of a single PCB by a vector network analyzer (solid line). The PCB was submerged in the suspension that was also used during the experiments (σ_m_ = 3 μS/cm). The dotted line shows the calculated absolute impedance of an ideal resistor ($407.7~\Omega$) in parallel to an ideal capacitance (4.1 nF). The values for resistor and capacitor are numerically derived from the measurement data. The yellow dashed line represents the calculated value for the case of an additionally ideal inductor (48 nH) in parallel to the ideal capacitor and resistor. Displayed frequency range is 10 kHz–15 MHz.

We developed high-throughput iDEP^22,43,44^ as well as eDEP^41,45^ DEP separators that are capable of processing up to 600 ml/h of sample volume. However, all of these separators suffer from a frequency limitation as the current exceeds the rating of the amplifier. The highest frequency we could apply was 500 kHz in an eDEP separator based on custom designed printed circuit boards (PCBs)^25^. Other researchers described similar problems. Farmehini et al.^46^ published an interesting report on the frequency limitations of amplifiers commonly used in DEP research and on the development of a novel amplifier. The authors link the frequency limitation to the maximum slew rate of an amplifier. Rabbani, Sonker, and Ros concluded in a review that one main limitation of iDEP separators in general is the low maximum frequency due to high applied potentials^3^. As in eDEP separators electrodes often are closer than in iDEP setups, sufficient field strength can be generated using lower potential^3^ which allows the application of higher frequencies. Finally, Boldt et al.^47^ investigated how the interplay of the output impedance of the amplifier and the impedance of the channel itself can have an impact on the manipulation of particles by DEP. The authors demonstrated that low electrical impedance of a channel can hinder particle manipulation by means of DEP. Here, not the slew rate but the electrical properties, namely its impedance, is limiting the performance of the device. Furthermore, a low impedance leads to high currents (Ohm’s law) that can exceed the specifications of the used amplifier, which is the reason for the frequency limitation of our eDEP separator based on PCBs. In fact, high power can be supplied for applications such as radio transmission. Several kW of output power can be achieved here. However, these amplifiers only work with very specific loads (e.g., 50 ohms pure ohmic resistance), which is usually not the case for DEP separators at high frequencies (see Fig. 2).

### The study

In this study, we aim to extend the frequency range of the aforementioned eDEP separator based on PCBs. The separator was characterized in previous studies^25,41,45^ where details can be found. Additionally, information is provided in the Methods section (“Methods” Section after the conclusion) of this article. The separator was originally designed to provide a low-cost alternative to increase the throughput of electrode-based DEP separators. Key element of the device are custom-designed PCBs that have an interdigitated electrode array (IDE) with a width and spacing of 250 μm patterned on them (Fig. 2A). Two identical PCBs are glued into two polypropylene holders that face each other and are separated by a 0.5 mm gasket which forms a channel between the two PCBs. The IDE produce a highly inhomogeneous electric field that results in DEP of the particles. A photograph of the assembled setup is provided in Supplementary Fig. 1 and 2. A particle suspension is entering the channel through an inlet and flows over the PCBs. Particles that show pDEP will be attracted toward the electrodes and may be trapped there. In contrast, particles that show nDEP are repelled from the PCBs and are eluted through the outlet. For this study, the layout of the PCBs was slightly altered compared to the previously presented design. The new PCBs are depicted in Fig. 2A and feature IDE on the front as before. The major modifications were the addition of solder pads on the back of the PCB to connect inductors to the device, the switch from for hot air solder leveling (HASL) to electroless nickel immersion gold (ENIG) cover layer of the electrodes to improve solderability and the usage of coaxial cable as connection to the PCB in contrast to regular stranded wire previously. Stranded wire is not suitable for high frequency application due to its parasitic properties and consequently was replaced for this study.

To understand the origin of the frequency limitation of a device, the impedance of the separator must be considered. In Fig. 2B, the absolute impedance of a single PCB glued into the setup is displayed. The data was recorded using a vector network analyzer (VNA) from 10 kHz to 15 MHz. In this frequency range, the electrical behavior of the device can be described by a resistor in parallel ($R=407.7$ $\Omega$) to a capacitor ($C=4.1$ nF) as can be seen by the fitted curve (Fig. 2, dotted line, calculations Supporting information chapter 2.1). Up to around 100 kHz, the impedance is almost constant but drops afterward. The decrease in impedance with increasing frequency indicates an increasing current. This current is linked to the capacitance of the device and the increasing displacement current. The energy input into the channel due to the displacement current is not dissipated by Joule heating, as it is the case for the free current^8^, but stored in the electric field. The separator acts similar to a capacitor at high frequencies. The stored energy is released afterward in the second half of an AC signal when the polarity switches.

Similar to a capacitor, an inductor can also store energy in one half cycle of an AC-signal and release it subsequently. Luckily, an ideal capacitor and an ideal inductor have a delay of one half cycle with respect to each other. Thus, they can form a resonant circuit where energy oscillates between an inductor and a capacitor. Once the oscillating circuit is fully loaded, it reduces the current drawn from the amplifier[^49^, p. 150 ff.]. This leads to an increase in the net impedance of the resonant circuit at specific frequencies. This operational procedure is called power factor correction (PFC) and is well-known in the field of electrical engineering. However, to the best of the authors’ knowledge, this was not applied in the field of DEP separators. This is probably due to the focus of DEP in microfluidic devices, where displacement currents often can be neglected^47^. As the size of the DEP separator increases, the capacitance of the device does come to a limiting factor as we noticed during our work (Eq. 3).

In this study, we demonstrate the applicability of PFC in high-throughput DEP devices to increase the applicable frequency range. With increasing the maximum possible frequency of the device, a step towards material-selective high-throughput separation via DEP is undertaken as broadening the frequency range allows to address different material combinations with DEP (Fig. 1).

We would like to note that as process engineers, electrical engineering is not the focus of our research. Consequently, we will focus on the DEP part of this project and use PFC as a tool. This study aims to act as starting point for future, more in depth analysis of this topic. We invite peers with other educational background to take this study as starting point for their research and help developing the combination of high-throughput DEP and PFC further. Even though the use of very high frequencies presented here makes it possible to penetrate into areas of highest separation efficiencies, we have—in contrast to earlier publications—not concentrated here on maximizing the separation efficiency of the filter. Instead, we focus on reporting the progress in terms of frequency bandwidth that can be achieved when using PFC.

## Results and discussion

Prior to applying PFC to our device, the impact of the modification compared to the previously published separator design was examined. As mentioned above, the modifications were the addition of solder pads on the back of the PCB to mount inductors, the switch from HASL to ENIG cover layer of the electrodes to improve solderability and the usage of coaxial cable as connection to the PCB in contrast to regular stranded wire previously. Additionally, a new amplifier that is designed for higher frequencies and voltages was used. For details on experimental setup and trapping experiments, please consult the Methods section of this publication (“Methods” Section after the conclusion).

### Comparison to previous filer design

**Figure 3:**
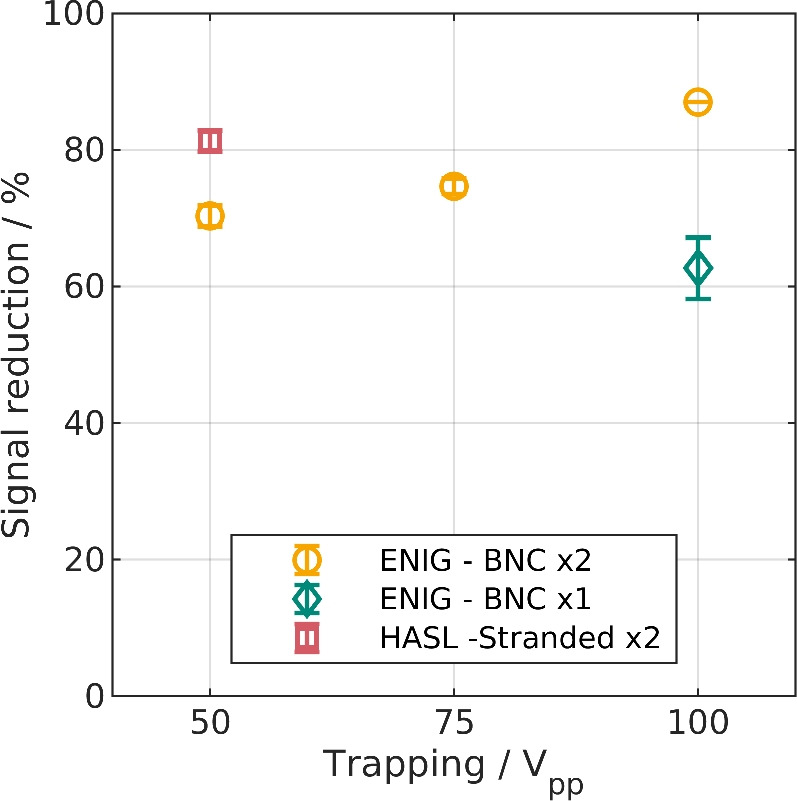
Results of the benchmark experiments of trapping KS6 particles at a flow rate of 6 ml/min, a medium conductivity of 3 μS/cm and a frequency of 500 kHz. The error bars indicate the standard deviation (n = 3). The HASL channel features stranded wire and is identical to the previously published setup in Ref.^25^. Additionally, the new setup with two PCBs connected to the amplifier (ENIG-BNC x2) and only one connected PCB (ENIG-BNC x1) are measured without any inductance soldered on the back of the connected PCBs.

To compare the new and the previously published designs, trapping experiments were carried out. Graphite particles (KS6) were suspended in an aqueous solution and pumped through the channel at a flow rate of 6 ml/min. We chose graphite particles for our experiments as no crossover is expected for these conducting particles. Consequently, the trapping of the particle can be used as an indicator for the magnitude of the electric field inside the channel. For comparison of the designs, a signal with a frequency of 500 kHz was applied, as this was the best performing frequency in a previous publication with these particles^25^. The removal of graphite during the application of the electric field is indicated by the signal reduction that was measured by an online reflection measurement. We chose to use the term signal reduction in contrast to trapping efficiency or separation efficiency, as the used graphite particles show a size distribution (*d*_50_ = 3.4 μm) and are irregular in shape. Consequently, the definition of trapping efficiency would be ambiguous as not all particles produce an equal reflection. Signal reduction, however, is a more intuitive parameter. A reduced signal is linked to fewer particles reaching the flow-cuvette and, thus, to particles being trapped inside the channel.

The results of these comparison experiments are visualized in Fig. 3. The reference case, which is the previously published^25,41^ PCB separator, produces at an applied voltage of 50$~\text{V}_\text{pp}$ ($\approx 18~\text{V}_\text{rms}$) a signal reduction of $81.3\%\pm 1.5\%$ (Fig. 3 red marker). This is slightly lower than reported in Ref.^25^ and might be linked to the higher medium conductivity in this study. We additionally checked whether the utilization of the new amplifier impacts the signal reduction. With the previously used amplifier (F30PV, Pendulum Instruments, Sweden) $83.0\%~\pm 1.0\%$ were achieved which indicates no significant influence of the amplifier itself.

The updated version of the setup, which features the above mentioned modifications shows lower signal reduction at $50~\mathrm {V_{pp}}$ when two PCBs are connected (Fig. 3 yellow markers). The values are $70.3\%\pm 1.5\%$, $74.7\%\pm 1.2\%$ and $87.0\%\pm 0.0\%$ at 50$~\text{V}_\text{pp}$, 75$~\text{V}_\text{pp}$ and 100$~\text{V}_\text{pp}$, respectively. A possible explanation could be that we used a simple $50~\Omega$ T-piece to split the signal from the amplifier and direct it to the two PCBs. According to the manufacturer of the amplifier, this should be avoided as this can produce reflections and thus lowering the effective voltage at the load (channel). Furthermore, the coaxial cables are designed to drive a restive load with an impedance of $50~\Omega$. Deviations from this optimum can cause signal loss. As we can see from Fig. 2B at 500 kHz the capacitive currents already have a significant impact on the impedance of the channel and the load has a more capacitive nature than purely resistive. Nonetheless, significant trapping is produced at $100~\text{V}_\text{pp}$ ($\approx 35~\text{V}_\text{rms}$) and the device functionality consequently could be validated.

Additionally, we measured the signal reduction with only one PCB of the new setup connected to the amplifier. The second PCB was disconnected from the amplifier but remained within the channel. This configuration becomes important in the following chapter, and consequently, we tested it without PFC as reference. As expected, the trapping efficiency is lower with only one PCB connected. Yet, $62.7\%\pm 4.5\%$ (Fig. 3 green marker) of signal reduction could be observed at $100~\text{V}_\text{pp}$ which is around three quarters of the value of two PCBs. This is higher than anticipated prior to the experiments. The disconnected PCB could act as floating electrode and could improve trapping as it can lead to additional field inhomogenities. We carried out electric field simulations that are shown in Supplementary Figs. 5 and 6. These simulations indicate the presence of additional field distortions due to the floating electrodes.

With these experiments, we could demonstrate the functionality of the modified setup. However, no higher frequencies than reported before were applied. When applying higher frequencies, we noticed a steep drop in separation efficiency (data is provided in the next section) and thus decided to perform PFC.

### Compensation of capacitive currents via inductors

To perform PFC, a suitable inductance connected in parallel to the channel is required. By using the measured electrical properties of the device ($R=407~\Omega$ and $C=4.1$ nF) one can calculate the required values of an inductor to compensate the capacitive currents at a specific frequency. For the proof-of-principle experiment, we aimed to achieve 10 MHz. This is 20-fold higher than the previously maximal frequency we could apply (500 kHz). Using these values, a required inductance of 61.8 nH is obtained. For details of the calculation, see “Calculating required inductance” Section. By soldering five inductors, each having an inductance of 240 nH, in parallel on the solder pads of the PCB, an effective inductance of $240~\text{nH}/5=48~\text{nH}$ can be obtained. While four of these inductors would already yield an effective inductance of 60 nH, we chose to add another one to securely exceed 10 MHz.

Inductors show low impedance at low frequency (Eq. 5). Consequently, adding an inductor in parallel to the device will yield a low impedance of the complete device in theory. This can be seen from the calculations shown in Fig. 2B (yellow line). Additionally, the calculations reveal that the impedance increases with frequency and a peak at 11.4 MHz can be seen. Here, currents through the inductor and the capacitor are expected to be equal in magnitude but phase-shifted and thus compensate each other. Afterward, the impedance is no longer dominated by the inductor but by the declining impedance of the capacitor. Finally, at frequencies of about 30 MHz both circuits (with and without an inductor in parallel) behave similar, as the inductor now has a high impedance and the overall impedance is dominated by the capacitance of the separator.

**Figure 4:**
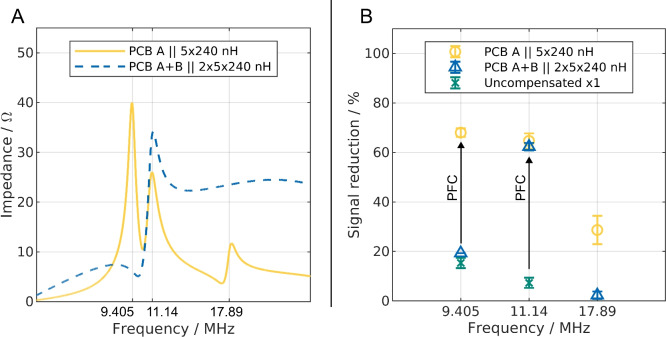
(**A**) Measured impedance using a vector network analyzer for PCB A individually and in combination with PCB B (A+B). (**B**) Results of the signal reduction at higher frequencies. PCB A and B have five 240 nH inductors soldered on their back. The yellow and green markers show results from experiments where only one PCB is connected to the amplifier. For the uncompensated case, however, no inductor is connected to the PCB. The blue marker display the results of two PCBs connected to the amplifier with a T-piece and each having five 240 nH inductors connected to them. The arrows indicate the increase in signal reduction due to the application of power-factor correction (PFC). Error bars indicate the standard deviation derived from three consecutive experiments. The experiments were conducted at a volume flow of 6 ml/min and a voltage of $100~\mathrm {V_{pp}}$.

In Fig. 4A, the measured impedance from 1 to 25 MHz of the device with inductors connected to it is shown. Displayed are , both, a single PCB named A (yellow line) and PCB A in combination with a second PCB named B after joining them with a T-piece (blue dashed line). The impedance of the single PCB B is provided in Supplementary Fig. 4. The impedance of PCB B does not deviate significantly from that of PCB A.

At 1 MHz, the absolute impedance is small for both displayed cases. This is, again, a result of the inductors now present in the electrical circuit. Both configurations in Fig. 4A show a peak of the impedance at around 11.1 MHz, which is close to the predicted value of 11.4 MHz. The difference could lie in the manufacturing tolerance of the inductors ($20~\%$). Consequently, we selected 11.14 MHz as the first frequency to conduct trapping experiments. Additionally, the single PCB A shows a peak at around 9.4 MHz and a smaller one at 17.89 MHz. The reason for the existence of these two additional peaks was not predicted by our calculations and is currently not entirely understood. These could be linked to non-ideal behavior of the inductors, the channel and the cables or an interplay of the aforementioned. The peak at 17.89 MHz is likely linked to parasitic influences (see “Compensation of capacitive currents via coaxial cable” Section). These two peaks are not present when both PCBs are simultaneously connected to the VNA. Nonetheless, we chose to test 17.89 MHz and 9.405 MHz in trapping experiments as well.

The results of the trapping experiments are displayed in Fig. 4B. Again, we tested the PCBs individually (yellow marker) and in combination (A+B, blue marker). The trapping of PCB B is very similar to that of PCB A (Supplementary Fig. 4). Additionally, we tested a single PCB with no inductor soldered (green marker in Fig. 4B) at 9.405 MHz and 11.14 MHz for comparison.

At 11.14 MHz both configurations with PFC show very similar performance. The mean values for the signal reduction are around $63\%$ at this frequency. Interestingly, connecting two PCBs does not improve the trapping compared to a single PCB. This differs compared to the experiments at 500 kHz (Fig. 3). A possible explanation is that connecting two PCBs by using two 50 $\Omega$ coaxial cable by a simple BNC T-piece to an amplifier could lead to reflections and thus lowering the effective voltage inside the channel. The amplifier is designed to drive $50~\Omega$ loads, connecting two coaxial cables with a design impedance of $50~\Omega$ each in parallel to two channels with about 25 $\Omega$ at 11.14 MHz each yields in a mismatch of the impedance which is known to cause reflections^48^. Furthermore, we would like to emphasize, that two PCBs normally are expected to perform better than a single PCB (e.g., Fig. 3). This result gives a hint that more insight into high frequency electronics and signal transmission are necessary to explain the performance difference between a single and two PCBs connected in parallel. The trapping of the uncompensated PCB is poor with only $7.3\%\pm 2.1\%$ at 11.14 MHz and clearly shows the benefit of using inductors for compensating high displacement currents. The channel impedance at that frequency is only a few ohms (see Fig. 2) and thus drastically lower than the output impedance of the amplifier. Boldt et. al. showed in 2021^47^ previously that low ohmic resistance due to high conductivity medium can reduce the effectiveness of DEP separators. This seems also to apply to low impedance due to high frequency in combination with significant capacity of the separator. In cases of low separator impedance, the voltage drop does not occur over the medium itself but over the output resistance of the amplifier.

At 9.405 MHz the two PCBs in combination showed no peak in impedance measurements. Here, we measured a significantly lower signal reduction of only 19.3% ± 3.1%. This is just above the measured value for the uncompensated PCB (15.3% ± 2.1%). Again, the low impedance of the channel seems to hinder particle trapping in both cases. In contrast, PCB A and B individually connected to the amplifier show similar but a bit higher signal reduction in comparison to 11.14 MHz (A: 68% ± 1.7%, B: 71% ± 0%). As the electrodes individually have an impedance peak at 9.405 MHz, this is in line with the previous results.

Finally, at 17.89 MHz, the trapping is poor compared to the other two frequencies for all configurations. Again, two PCBs in parallel are performing worse than the single PCBs. Here, the impedance cannot explain the difference, as it is higher for the two PCBs in parallel. However, no distinct impedance peak is visible in contrast to the single PCB. Despite the fact that the reason is currently not entirely understood, it is possible to identify promising frequencies with VNA measurements relatively straight-forward.

From the presented results, we draw multiple conclusions. First, preferably only one PCB should be used in high-frequency high-throughput DEP as it simplifies the design and can even increase separation efficiency. Second, these results indicate the potential of PFC in DEP. Third, they also show the necessity of taking the high-frequency behavior of not only the separator but also the other components, such as the amplifier and coaxial cable, into account. This study shows, consequently, the requirement for interdisciplinary research once more. Existing concepts from other fields of research (e.g., antenna layout/signal transmission) could be highly beneficial for the progress of high frequency DEP especially for larger separators.

**Figure 5:**
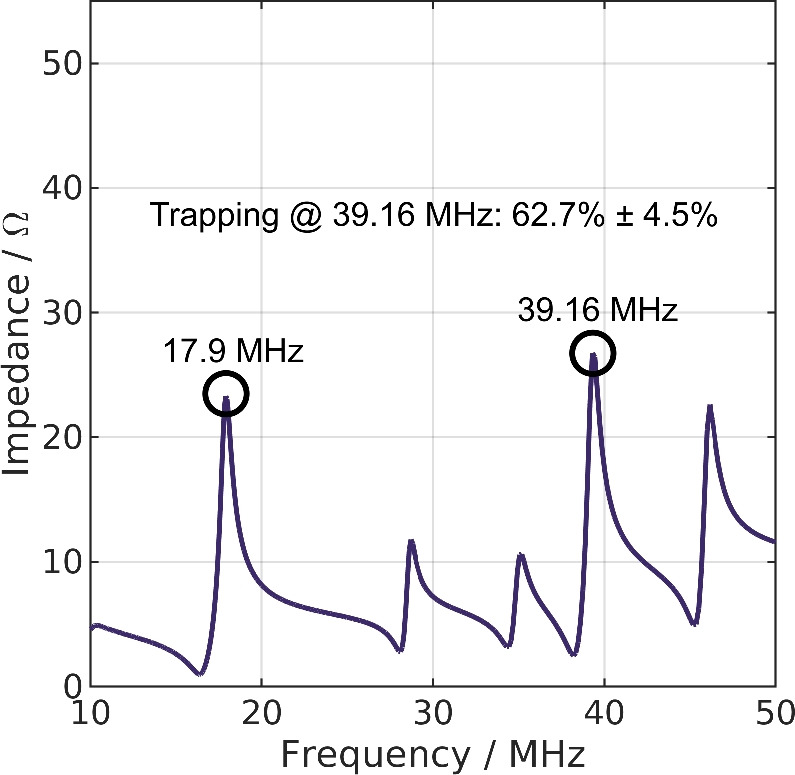
Impedance measured with a vector network analyzer of a single printed circuit board (PCB) with no inductors soldered to the back of the PCB. An around 1.5 m long soldered coaxial cable is connected to the channel. The data is the continuation of the measurement data displayed in Fig. 1. No inductors are soldered to the back for the compensation of capacitive currents. The frequency used in the experiment is highlighted. The trapping rate at 39.16 MHz (6 ml/min, 3 μS/cm) is displayed in the figure.

### Compensation of capacitive currents via coaxial cable

In addition to the deterministic way of PFC via soldering inductors to the electrodes, we found, that there is also a less deterministic way of PFC. In Fig. 5, the continuation of the impedance versus frequency measurement of a DEP separator featuring only one uncompensated PCB is provided. We found multiple peaks at frequencies over 10 MHz that are likely linked to the parasitic inductance of the coaxial cables and the PCB itself. One peak of impedance was observed at 17.9 MHz. At his frequency, a peak was also measured for the PCBs with inductors soldered on their back (Fig. 4A). We assume that this is consequently linked to the parasitic properties of the cable and the PCB.

Despite being found by chance and not deterministic, we decided to include these results for completeness. The highest absolute impedance within this frequency spectrum was found to be nearly $30~\Omega$ at 39.16 MHz. We conducted experiments at 39.16 MHz at 6 ml/min. We found the signal reduction to be $62.7\%\pm 4.5\%$. Please note that we were not able to verify the applied voltage with an external device at this frequency. However, the voltage monitor of the amplifier indicated a voltage of $100~\mathrm {V_{pp}}$ (details, see “Experimental setup” Section). Similar to the deterministic tuning of the impedance described in “Compensation of capacitive currents via inductors” Section, an increase of the impedance due to the parasitic properties of the setups allows trapping of particles inside the channel.

## Conclusion

In the study, successful compensation of capacitive currents via PFC was shown. The presented method allows entering the megahertz frequency range for a high-throughput DEP separator based on printed circuit boards for the first time. For this, we used low-cost components (VNA and inductors) to significantly enlarge the frequency bandwidth of the separator. A minimalist approach was chosen by simply connecting multiple inductors in parallel to the channel. However, more elaborate schemes exist, as PFC is an established tool in electrical engineering. Furthermore, impedance matching by designing the electrical properties of the channel by changing electrode geometries and/or channel size in combination with impedance matching networks should be considered in the future.

This report does not focus on maximizing the trapping efficiency but on introducing PFC as a tool for high-frequency and high-throughput DEP and should be seen as a proof-of-principle. Trapping efficiencies could be increased by higher voltages or lower superficial velocity via decreasing the flow rate or increasing the channel width. The tuning of trapping rates was extensively studied in the past^22,23,25,45^. Prior to focusing on increasing trapping efficiency, future studies should investigate the high-frequency behavior, namely impedance, phase shift and reflections of the separator and the interconnections used, in greater detail.

The presented results confirm the applicability of PFC in DEP separators and may pave the way toward separation of semiconducting particles using DEP at high volume flows. Additionally, a less deterministic way for PFC was presented that only featured the separator itself and a coaxial cable.

## Methods

### Fabrication of the channel

The setup is similar to the previously published DEP separator based on PCBs^25,45^. A photograph of the assembled setup can be found in Supplementary Figs. 1 and 2. The design of the PCBs was updated for this study. At the back, solder pads for coaxial cable and inductors were added. Additionally, more interlayer connections (vias) were included to distribute the current flowing from the front of the PCB to the back and vice versa over multiple vias and thus a larger area. The custom-designed PCBs were produced externally (JLCPCB JiaLIChing (Hong Kong) Co. Ltd., China).

The PCBs are $45\times 150$ mm in size and have an interdigitated electrode array on their front with 145 pairs of electrodes each. The width and the distance of the electrodes are 250 μm. In contrast to previous publications, the copper electrodes on the PCB are not covered with lead-free hot air solder leveling (HASL) but with electroless nickel immersion gold (ENIG) to improve solderability. Two identical polypropylene holders are used into which the PCBs are glued. The two PCBs are facing each other and are separated by a 0.5 mm silicone gasket.

To connect the channel to the amplifier (or VNA) we soldered a standard $50~\Omega$ (RG 58C/U SYTRONIC, reichelt elektronik, Germany) coaxial cable to the PCB. To the other end of the cable, a BNC-connector (0401256/D, BKL-Electronic, reichelt elektronik, Germany) was crimped. The coaxial cable had a length of 1.5 m. For PFC, we selected shielded inductors with an inductance of 240 nH ($\pm \,20\%$) (DFE201610P-R24M=P2, Murata Electronics, Mouser, Germany) that were also soldered to the back of the PCB. By changing the number of inductors soldered to the PCB, the effective inductance can be chosen.

### VNA measurements

We used a standard open load (SOL) calibration to calibrate the VNA (DG8SAQ USB-Controlled VNWA 3,SDR-Kits, United Kingdom) prior to the experiments. The VNA and the calibration standards are depicted in Supplementary Fig. 3. During calibration, we connected a coaxial cable (1.5 m length, identical to those soldered to the PCBs) to connect the VNA to the BNC calibration standards to account for the cable of the separator. Additionally, a $50~\Omega$ T-piece was used to enable connection between the calibration standards and the coaxial cable. When changing the frequency range, we renewed the calibration. Each measurement contains 2500 data points, and a measurement time per data point of 6 ms was selected, totaling a duration of 15 s per measurement. All VNA measurements were conducted while pumping particle suspension through the channel.

The data measured by the VNA was used to determine the frequencies were trapping experiments in the channel should be tested. Furthermore, the existence of the peaks in the impedance spectrum (Fig. 4A) themselves indicated whether the compensation of capacitive currents was at least partially successful. However, when evaluating the VNA measurements one needs be to careful, as the measurement method is sensitive to calibration errors. As the coaxial cable is soldered to the PCB, and, during calibration, an additional T-piece was used to connect the standards, the data should be interpreted carefully. Furthermore, the oscillating circuit showed in LT-spice simulations (data not shown) that it takes some full cycles of the AC signal until a steady-state is reached. Currently, we are not sure how this affects the data obtained from the VNA and whether this could be one reason why the impedance of the compensated PCBs does not reach the DC-resistance of the device. This should be clarified in follow-up studies. Last, we did the calibration of the VNA with only a single coaxial cable, despite the fact that two cables are required to connect both electrodes simultaneously. Consequently, we only extracted the position of the impedance peaks from the VNA measurements and did not discuss nor present the measured phase shift of the signal. Nonetheless, the location of the peaks is the crucial parameter for our experiments, and the VNA has proven its value for this task.

### Calculating required inductance

To find the correct inductor for a certain frequency, the electrical capacitance of the device must be known. The impedance of an ideal capacitor $Z_\text{C}$ can be calculated with^42^

4 $$\begin{aligned} Z_\text{C}=\frac{1}{j\omega C}, \end{aligned}$$

where *j* is the imaginary unit and C the capacitance. The impedance of an ideal inductor $Z_\text{L}$ in contrast follows the equation^42^

5 $$\begin{aligned} Z_\text{L}=j\omega L. \end{aligned}$$

whit *L* being the inductance. Comparing these two equations, it becomes obvious that the impedance of a capacitor decreases with frequency while it increases for inductors. For effective PFC, currents in the capacitor and in the inductor are required to be of the same magnitude. Please note that between these currents, a phase shift is present, and inductor and capacitor (channel) can form an oscillating circuit. For a target frequency of 10 MHz one can calculate:^49^, p. 150ff.

6 $$\begin{aligned} | Z_\text{C}|&{\mathop {=}\limits ^{!}} |Z_\text{L}| \end{aligned}$$

7 $$\begin{aligned} L&=\frac{1}{\omega ^2C} \nonumber \\ L&=\frac{1}{(2\pi \cdot 10~\text{MHz})^2 \cdot 4.1~\text{nF}}=61.8~\text{nH} \end{aligned}$$

Inductors can be purchased in a broad variety in size, inductance and power ratings. To choose the correct inductor, the current rating of the inductor needs to be considered. The displacement current $\textbf{D}$ for one PCB at 10 MHz and 100 $\mathrm {V_{pp}}$ (35 $\mathrm {V_{rms}})$ is expected to be:

$$\begin{aligned} \textbf{D}=\frac{\text{Voltage}}{Z_\text{C}}=\frac{35~\mathrm {V_{rms}}}{\frac{1}{2\pi \cdot 10~\text{MHz}\cdot 4.1\mathrm {~nF}}} =9~\mathrm {A_{rms}}. \end{aligned}$$

Consequently, when selecting an inductance, it must be capable of handling $9~\mathrm {A_{rms}}$. The displacement current can easily exceed the current rating of a single inductor. However, parallel circuits of inductors can distribute the current and help to adjust the net inductance to the desired value.

### Experimental setup

The measurement setup for measuring the reflection signal is identical to previous publications^45^. The outlet of the separator is directed into the inlet of a flow cuvette (176-765-14485-40, Hellma GmbH & Co. KG, Germany). To a cuvette holder (CVH100/M, Thorlabs GmbH, Germany) a white-light source (XCite 120 PC, Excelitas Technologies Corp., USA) is connected by a liquid light guide. A spectrometer (Silver nova, StellarNet, Inc., USA) measures reflection intensity from 190 to 1100 nm during experiments. The light from the flow cuvette reaches the spectrometer through a light guide behind a triple-bandpass filter (DAPI/FITC/TRITC). Using software (Labview), the measurement intensity over time is stored in data files.

The particle suspension contained 10 mg/L Timrex KS6 (MSE Supplies LLC, USA). The particles are distributed in shape and size (*d*_50_ = 3.4 μm, manufacturer information). The particles were suspended in a low conductivity aqueous solution (σ_m_ = 3 μS/cm) that contained pure water (Omniatap 6 UV/UF, stakpure GmbH, Germany), $0.01\%$ Tween 20 (Sigma-Aldrich, Germany) as detergent, KCl to reach the desired conductivity, and 10 μM KOH to adjust pH. The particle suspension was pumped through the channel by a piston pump (MFLX78018-60, VWR International GmbH, Germany). Prior to the experiments, we degassed the medium by stirring it at a pressure of 70 mbar for several minutes. We continued to stir the suspension during experiments to avoid sedimentation.

The sinodial voltage signal was generated by using a signal generator (Rigol DG4062, Rigol Technologies EU GmbH, Germany) and amplified by an amplifier (A 1020-75-400, Dr. Hubert GmbH, Germany). The applied voltage was monitored by using the voltage monitor output from the amplifier and an oscilloscope (Rigol DS2072A, Rigol Technologies EU GmbH, Germany). Prior to experiments, we connected a T-piece at the power output of the amplifier and checked the monitor value versus the signal from the output using oscilloscopes. We were able to verify the output voltage up to 10 MHz. At higher frequencies, we were not able to verify the voltage at that frequency using this protocol. We do not recommend this procedure, nor does the manufacturer of the amplifier, as it lead to damage to two of our oscilloscopes at higher frequencies than 10 MHz. Consequently, in the future, additional equipment to monitor the signal needs to be considered. For higher frequencies, we assumed that the output from the voltage monitor remained constant compared to the generated voltage.

Prior to experiments with particles, the background was measured and subtracted from the measurement data. During trapping experiments particle suspension was constantly entering the channel. The initial reflection signal was monitored for 30 s at the beginning of each experiment to get the initial concentration of particles. Afterward the voltage was turned on for 270 s. In this period, particle trapping was expected. Subsequently, the voltage was turned off and previously trapped particles were released. The signal was then monitored until at least 600 s in total. We evaluated the stored data files with in-house-developed Matlab scripts. The data was smoothed by a moving average algorithm. All displayed data and the required evaluation scripts were uploaded to an online repository for public access^50^.

### Supplementary Information

Supplementary Information.

## Data Availability

All measurement data that is included in this publication is uploaded to an online repository^50^ along with MATLAB scripts to evaluate the data.
