# Supplementary material for: Compensation of capacitive currents in high-throughput dielectrophoretic separators
Source: Sci Rep. 2024 Jul 17;14:16491. doi: 10.1038/s41598-024-67030-9 (PMC11255223; doi:10.1038/s41598-024-67030-9)
Supplement: Supplementary file 1 — Supplementary Information. [file 41598_2024_67030_MOESM1_ESM.pdf]

Supporting information
Compensation of capacitive currents in high-throughput dielectrophoretic separators

Jasper Giesler[1], Laura Weirauch[1], Jorg Thöming[1,2,3], and Michael Baune[1,3,*]

[1]Chemical Process Engineering, Faculty of Production Engineering, University of Bremen, Leobener Straße 6,28359 Bremen,Germany
[2]MAPEX Center for Materials and Processes, University of Bremen, 28359 Bremen, Germany
[3]Center for Environmental Research and Sustainable Technology (UFT), University of Bremen, Leobener Straße 6, 28359 Bremen
[*]mbaune@uni-bremen.de

ABSTRACT

Separation and classification are important operations in particle technology, but they are still limited in terms of suspended particles in the micrometer and nanometer size-range. Electrical fields can be beneficial for sorting such particles according to material properties. A mechanism based on strong and inhomogeneous fields is dielectrophoresis (DEP). It can be used to separate microparticles according to their material properties, such as conductivity and permittivity, by selectively trapping one particle type while the other can pass the separator. Conventional DEP-separators show either a limitation in throughput or frequency bandwidth. A low throughput limits the economical feasibility in many cases. A lower frequency bandwidth limits the variety of materials that can be sorted by DEP. To separate semiconducting particles from a mixture containing particles with higher conductivity according to their material, high frequencies are required. Possible applications are the separation of semiconducting and metallic carbon nanotubes or the separation of carbon-coated lithium iron phosphate particles from graphite in the recycling process of spent lithium-ion batteries. In this publication, we aim to display how to tune the electrical impedance of a high-throughput DEP separator based on custom-designed printed circuit boards to increase its frequency bandwidth. By adding inductors to the electrical circuit, we were able to increase the frequency bandwidth from 500 kHz to over 11 MHz. The experiments in this study act as proof-of-principle. Furthermore, a non-deterministic way to increase the impedance of the setup is shown, yielding a maximum frequency of 39.16 MHz.

1 Photographs of the equipment

Additionally, to the photographs of the PCB in the main document, three more pictures are provided. In Figure 1 the assembled is shown. In the picture coaxial cables and tubing are included. The coaxial cable are soldered to the PCB by removing the isolation from the cable and soldering the core to one pad and the shielding to the other. To reduce stress on the solder joint, hot glue was poured onto the connection. In Figure 2 a close-up of a build-in PCB is shown. Four inductors can be seen in this photograph. At a closer look, the PCB tracks, covered with the solder mask of the PCB can be seen. The PCB tracks connect the inductors to the coaxial cable and the interlayer connectors.

In Figure 3 the VNA including the calibration standards and a 50 Ω T-piece for BNC are displayed. Despite only being a measurement device, we decided to include a picture of the VNA as it is, until now, uncommon in the area of DEP research.

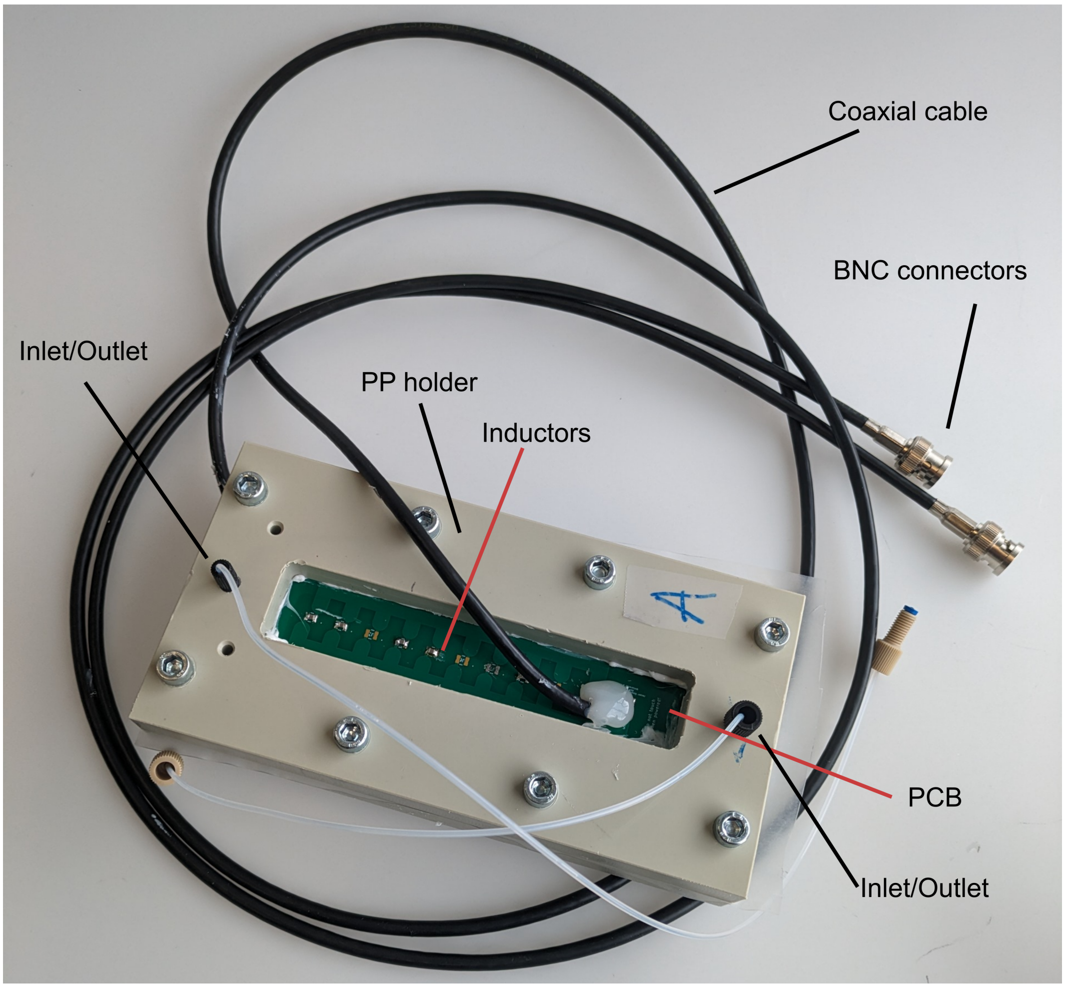

Supplementary figure 1. Photograph of the assembled setup including coaxial cable, and tubing through which the particle suspension is pumped.

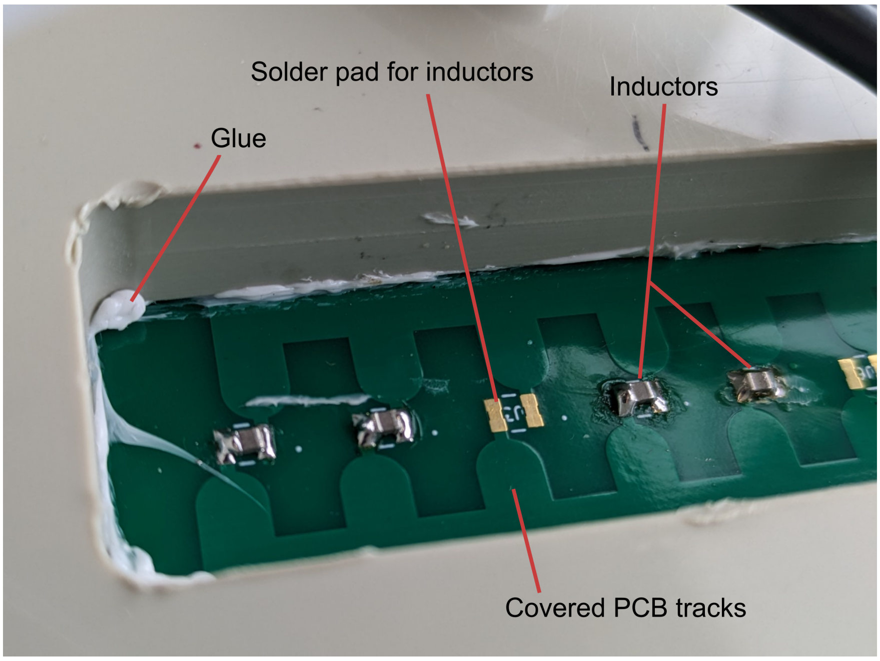

Supplementary figure 2. Close-up photograph of four inductors soldered to a PCB.

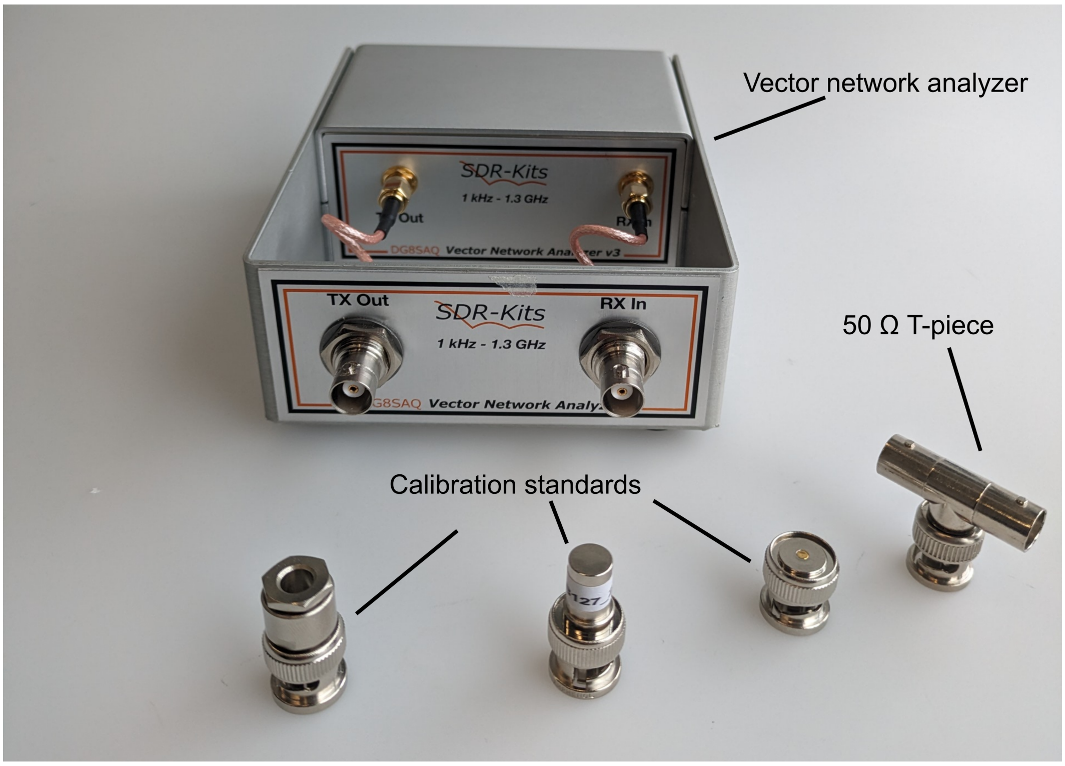

Supplementary figure 3. Photograph of the used VNA with the SOL calibration standards and a 50 Ω T-piece used in this study.

2 Additional data

2.1 Fitting the RC-circuit

In Figure 2 (main document), a calculated impedance of a ideal RC-circuit is shown. A RC-circuits consists of a capacitor with a capacitance C in parallel to a resistor with a resistance R. Its overall Impedance Z_{tot} depends on the angular frequency of the signal ω and can be calculated with [1, p. 140ff.]

$$Z_{\text{tot}} = \left(\frac{1}{R} + j\omega C\right)^{-1}. \tag{1}$$

We used a Matlab build-in optimization function to find the best fit for our measured data of the impedance. The script can be found in the online repository of this publication.

2.2 Electrode B

In Supplementary figure 4, the data of Figure 4 of the main document is again displayed. Additionally, the data for PCB B is included.

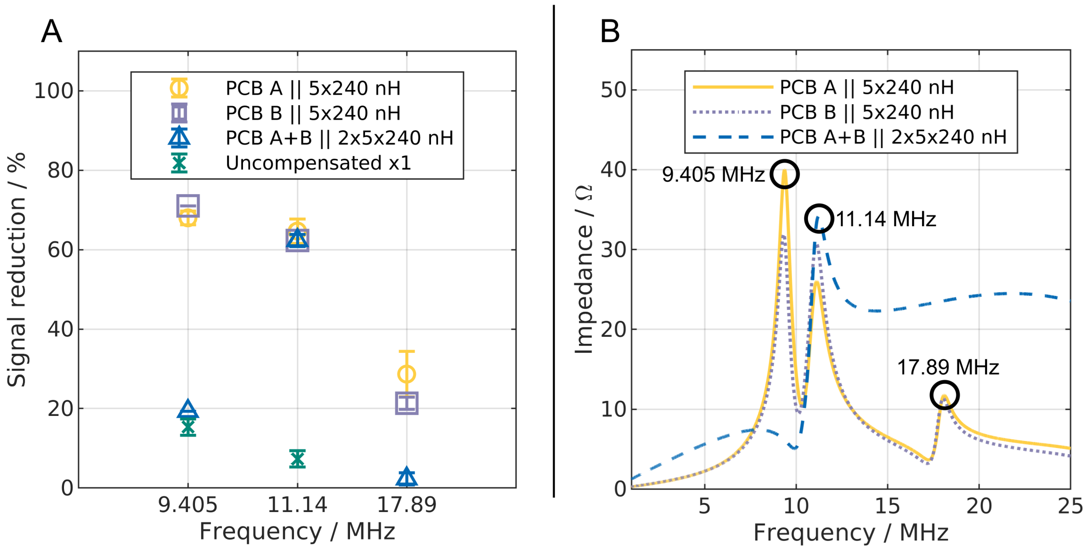

Supplementary figure 4. A: Results of the trapping rates at higher frequencies. Two single PCBs with five 240 nH inductors each soldered on the back (PCB A and B). Here, only one PCB is connected to the amplifier at a time. This is also true for the uncompensated case where no inductor is connected to the PCB. Additionally, results of both PCBs (A+B, blue marker) connected to the amplifier with a T-piece and in sum ten inductors. Error bars indicate the standard deviation derived from three consecutive experiments. The experiments were conducted at a volume flow of 6 ml/min and a voltage of 100 V_{pp}. B: Measured impedance with using a vector network analyzer for PCBs A and B individually and in combination (A+B, blue line). The used frequencies in the experiments in part A of the figure are highlighted.

2.3 Simulation of electric field

Electric field simulations were carried out using Comsol Multiphysics and its electric currents interface. Instead of simulating the entire channel, a representative 2D part of the channel was selected. The boundary conditions are visible in Supplementary figure 5. To the electrodes on the top, no fixed voltage boundary condition was applied. Thus, the simulations represent the case where these electrodes act as floating electrodes.

In Supplementary figure 6 the norm of the simulated electric field is provided. Clear maxima are developed at the electrodes that have a fixed voltage boundary condition applied to them. In contrast, in Supplementary figure 6A no distinct maxima are visible for the floating electrodes. However, when looking at part B of the same figure, some additional field distortions are revealed around the floating electrodes. In part B of the figure, the maximum of the color bar is lowered. These distortions will lead to an additional movement of the particles due to DEP. However, compared to the maximum of the electrodes at the bottom, their influence will be smaller.

References

1. Hüning, F. The Fundamentals of Electrical Engineering: For Mechatronics. In *The Fundamentals of Electrical Engineering*, DOI: 10.1524/9783110349900 (De Gruyter Oldenbourg, 2014).

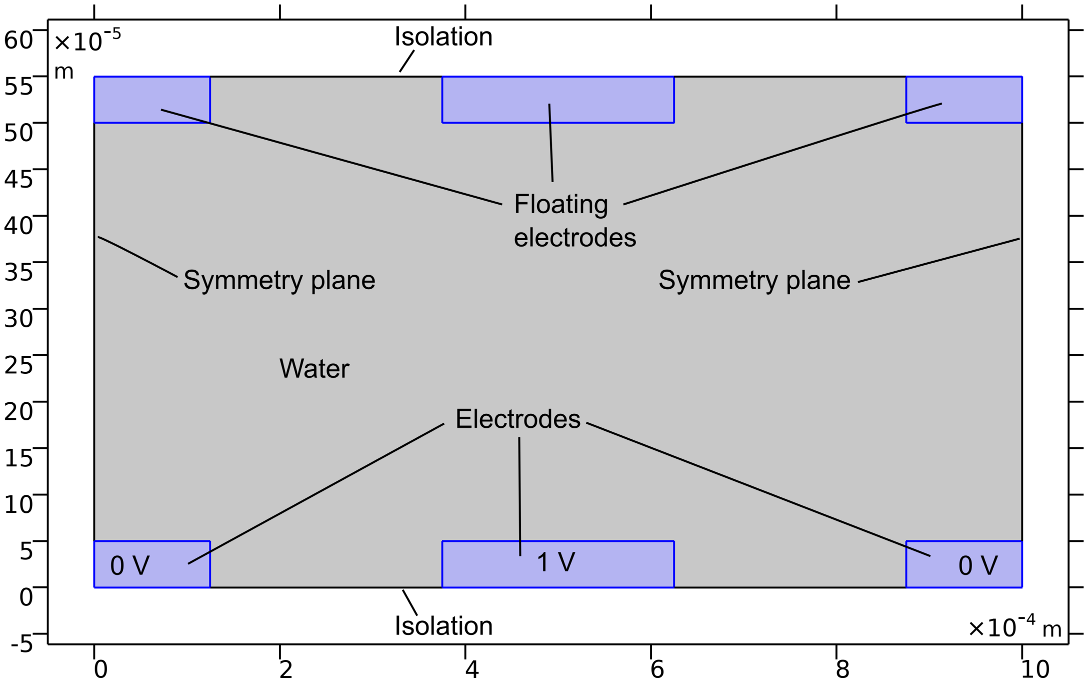

Supplementary figure 5. Representation of the simulated part of the channel and the used boundary conditions.

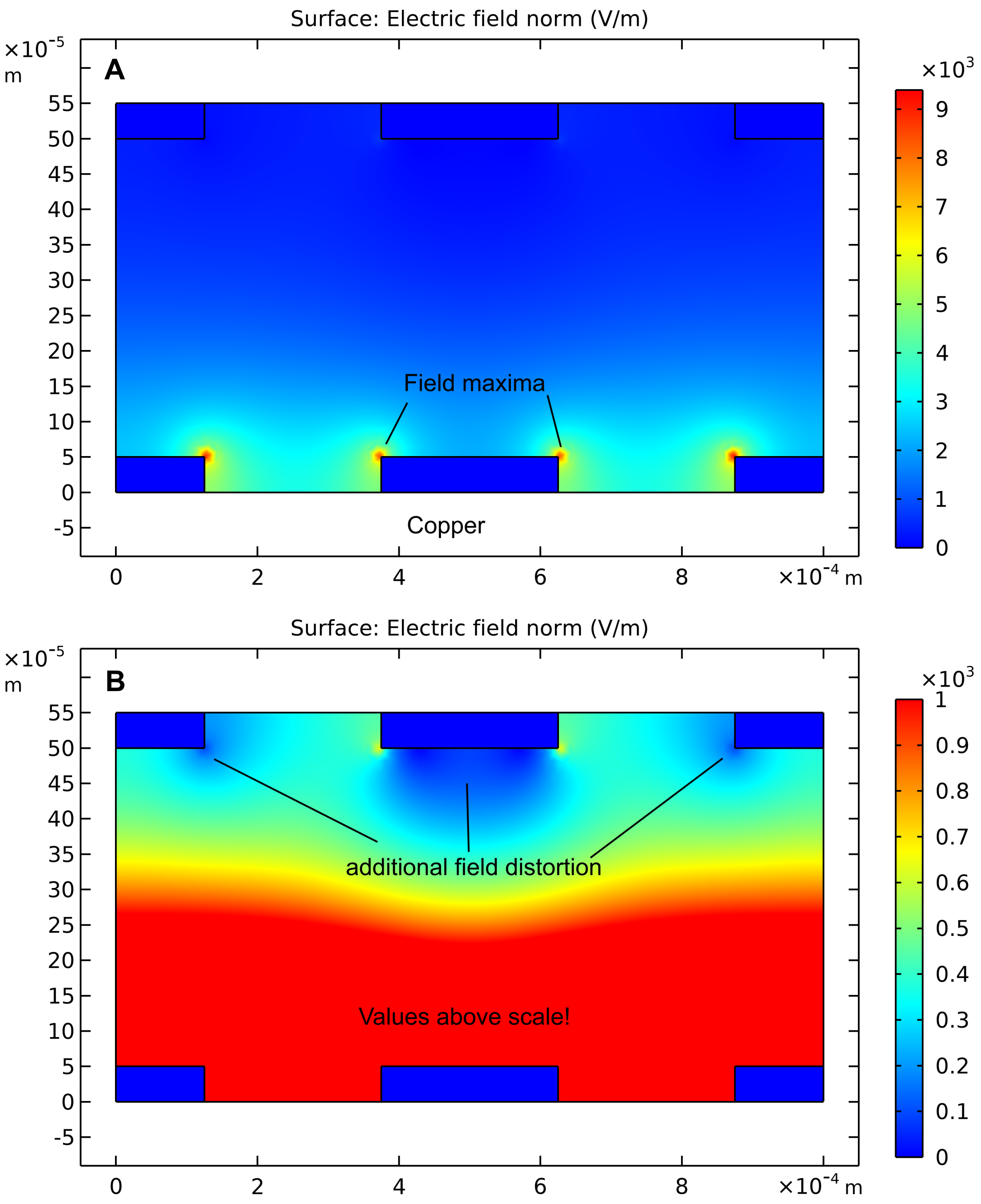

Supplementary figure 6. Simulated norm of the electric field. Part A and B of the figure display the same dataset, however, different scales of the colorbars are plotted. The applied boundary conditions are provided in Supplementary figure 5.
